# Incidence and recurrence rate of sigmoid diverticulitis in patients requiring admission to hospital in Iceland from 1985 to 2014: nationwide population‐based register study

**DOI:** 10.1002/bjs5.50336

**Published:** 2020-09-09

**Authors:** B. T. Alexandersson, T. Stefánsson

**Affiliations:** ^1^ Department of Internal Medicine, Section of Gastroenterology and Hepatology Reykjavik Iceland; ^2^ Department of Surgery The National University Hospital of Iceland Reykjavik Iceland

## Abstract

**Background:**

Diverticulitis is the most common complication of diverticular disease, affecting 10–25 per cent of patients with diverticula. A retrospective, nationwide, population‐based cohort study was performed to analyse the incidence and recurrence rate of sigmoid diverticulitis requiring hospital admission.

**Methods:**

All patients discharged from hospital in Iceland during 1985–2014 who were diagnosed with diverticular disease were included. The χ^2^ test was used to analyse the trend of the incidence in the period 2002–2014. The Kaplan–Meier method and the Cox model were used to analyse recurrence.

**Results:**

Of 8660 admissions for diverticular disease, 4746 were due to diverticulitis, of which 2939 were for diverticulitis diagnosed for the first time. After the first attack, surgery was used to treat 661 patients. Of 2278 patients not treated by resection, 537 had a second attack (23·6 per cent). There was a significant decrease in the incidence of diverticulitis in patients aged 40–89 years during the period from 2002 to 2014 (*P* = 0·033). The risk of recurrence was associated with younger age at first attack and female sex (*P* < 0·001).

**Conclusion:**

There was a decline in the incidence of patients hospitalized with diverticulitis between 1995 and 2014, most prominent in older age groups. Different recurrence rates were reported in men and women, and in younger compared with older age groups.

## Graphical abstract

Has there been an epidemic of sigmoid diverticulitis in Iceland, with the highest incidence around 1995? Changes in the prevalence of smoking, differences in diagnostic methods and improved health in older generations are possible causes of the rise and fall in incidence of sigmoid diverticulitis. Different recurrence rates point to different aetiological factors in younger and older patients, and in men and women.

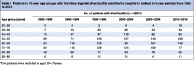

## Abstract (es)

**Antecedentes:**

La diverticulitis es la complicación más frecuente de la enfermedad diverticular y afecta al 10‐25% de los pacientes con divertículos. Se realizó un estudio de cohortes retrospectivo de base poblacional a nivel nacional para analizar la incidencia y la tasa de recidiva de pacientes con diverticulitis sigmoidea que requieren ingreso hospitalario.

**Métodos:**

Se incluyeron todos los pacientes dados de alta hospitalaria en Islandia durante el período de estudio entre 1985‐2014 que fueron diagnosticados de enfermedad diverticular. Se utilizó el método de ji al cuadrado para analizar la tendencia de la incidencia en el período 2002‐2014, y los métodos de Kaplan Meier y el modelo de Cox para analizar las recidivas.

**Resultados:**

De 8.660 ingresos por enfermedad diverticular, 4.746 se debieron a diverticulitis, de los cuales 2.939 correspondían a un primer diagnóstico de diverticulitis. Después del primer episodio, se intervinieron 661 pacientes. De 2.278 pacientes que fueron tratados sin resección, 537 presentaron un segundo episodio (24%). Hubo una disminución significativa en la incidencia de diverticulitis en pacientes de 40 a 89 años durante el período 2002‐2014 (*P* = 0,033). El riesgo de recidiva se asoció con una edad más joven en el primer episodio y con el sexo femenino (*P* < 0,001).

**Conclusión:**

Se observó una disminución en la incidencia de diverticulitis que precisó hospitalización de 1995 a 2014, siendo más notoria en los grupos de mayor edad. Se observaron diferencias en las tasas de recidiva en los varones *versus* las mujeres y en los grupos de edad más joven *versus* los de mayor edad.

## Introduction

The prevalence of diverticular disease increases with age, being reported as less than 10 per cent in people younger than 40 years and up to 66 per cent in patients older than 80 years, with similar rates in the female and male population^1^. Diverticulitis, an inflammation or infection in a diverticulum, is usually located in the sigmoid colon, and can occur in 10–25 per cent of these patients^2^, ^3^. Complications of diverticulitis include colonic perforation or intra‐abdominal infection or abscess, and are usually diagnosed by clinical examination (pain and tenderness in the lower abdomen combined with fever), laboratory tests (raised white blood cell count or C‐reactive protein level) and imaging (CT or ultrasound imaging). The causes of diverticulosis are genetic and age‐related^4^, probably owing to changes in the neuromuscular function and decreased strength in the connective tissue^5^, whereas the development of diverticulitis is related also to sex, immunosuppressive therapy, administration of steroids and non‐steroidal anti‐inflammatory drugs, and smoking^5^, ^6^, ^7^, ^8^, ^9^.

**Fig. 1:**
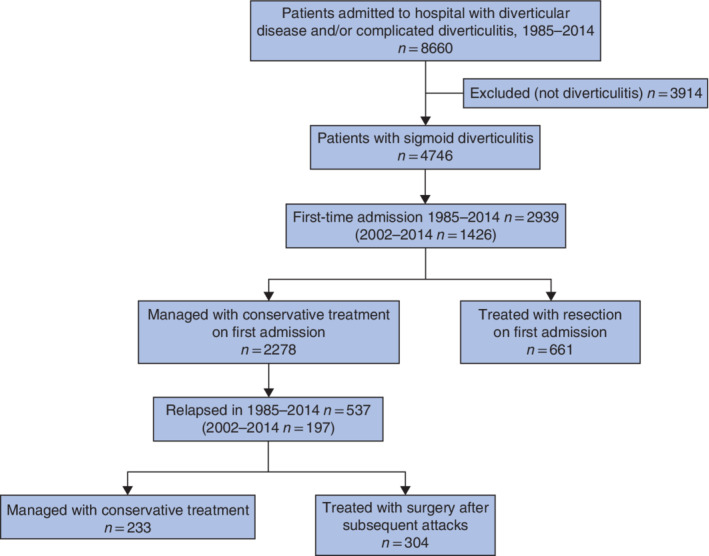
Flow diagram for the diagnosis of included patients and referral for surgery

ICD‐9 and ICD‐10 differentiated diverticular disease (which includes diverticulosis and uncomplicated diverticulitis) and complicated diverticulitis, but did not use specific codes for uncomplicated disease. Accordingly, it is difficult to study the admission rate and incidence of uncomplicated sigmoid diverticulitis, except in Denmark, where a specific code is used. A population‐based study in Norway^10^ showed an increased incidence of sigmoid diverticulitis between 1988 and 2012, similar to findings in a Danish population‐based study^11^. An increased incidence of diverticular disease has also been reported in the USA (2000–2007 *versus* 1980–1989)^6^, England (1999–2000 *versus* 1989–1990)^12^ and Scotland (2000–2010)^13^. However, a recent study^14^ on diverticular disease in Sweden documented that between 1997 and 2010 the incidence did not increase, and in the population aged over 70 years there was a small but significant decrease in the incidence of diverticular disease. On this basis, the aim of this study was to analyse the incidence and recurrence of sigmoid diverticulitis requiring admission to hospital in Iceland from 1985 to 2014.

## Methods

This research was designed as a retrospective population‐based and nationwide cohort study. All patients

**Fig. 2:**
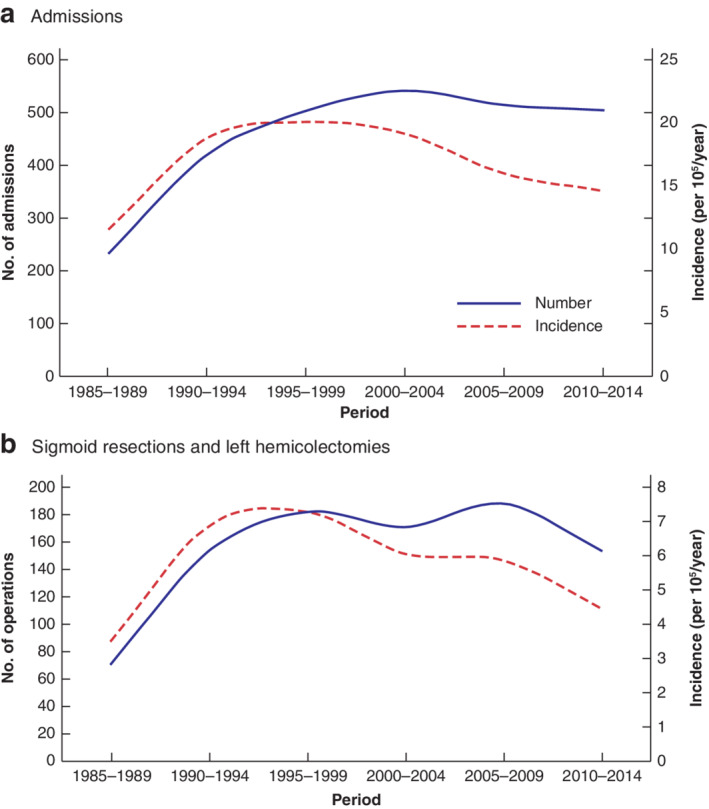
Number and incidence of admissions to hospital and of sigmoid resections and left hemicolectomies for sigmoid diverticulitis in patients aged 40–89 years in 5‐year periods from 1985 to 2014 **a** Admissions to hospital; **b** sigmoid resections and left hemicolectomies.

**Fig. 3:**
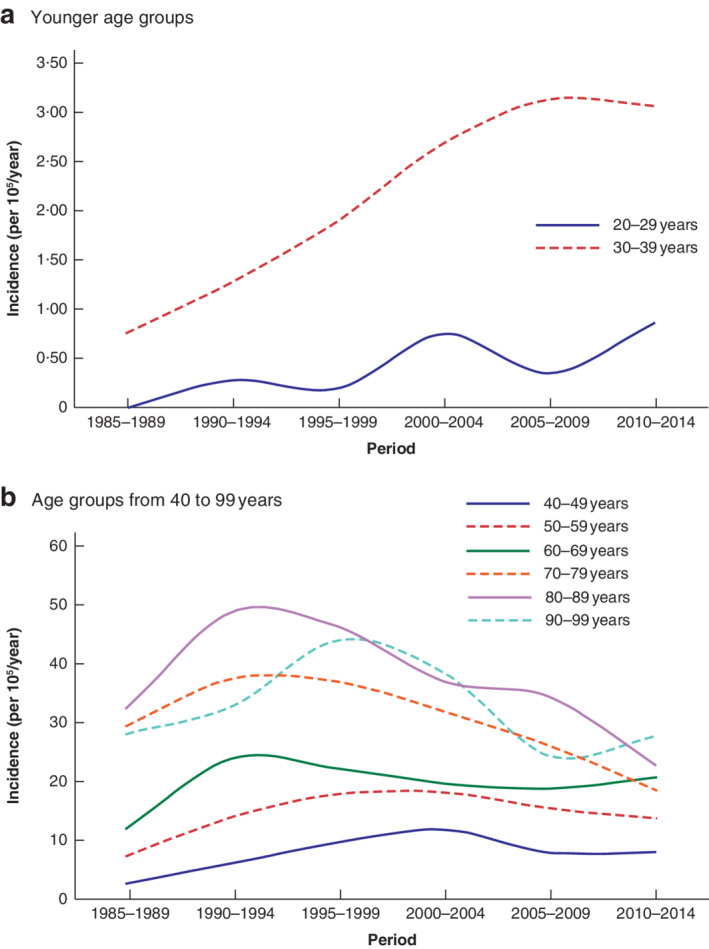
Incidence of admissions for sigmoid diverticulitis in 10‐year age groups for 5‐year periods from 1985 to 2014 **a** Age groups 20–29 and 30–39 years; **b** 10‐year age groups from 40 to 99 years.

discharged with the diagnosis of diverticular disease and complicated diverticulitis (ICD‐9 diagnosis codes 562.1 and 562.11 (1985–1998) and ICD‐10 diagnosis codes K57.2, K57.3, K57.4, K57.5, K57.8, K57.9 (1999–2014)) from The National University Hospital of Iceland in Reykjavik and Akureyri Hospital in northern Iceland during 1985–2014 were included. These two hospitals cover almost the whole country and treat all patients with diverticulitis requiring surgery. All Icelandic citizens have good access to primary healthcare and to the hospitals' emergency departments, and their records can be identified using a ten‐digit national registration number, a unique personal identifier assigned to all citizens.

The hospital statistics were retrieved from an Excel® (Microsoft, Redmond, Washington, USA) sheet containing standard information for each patient. As well as the national registration number, each patient record contains data on hospital department, surgical procedures and discharge diagnosis, in which the first diagnosis is the most important cause of admission. These diagnoses were coded according to ICD‐9 until 1996 and ICD‐10 thereafter.

**Fig. 4:**
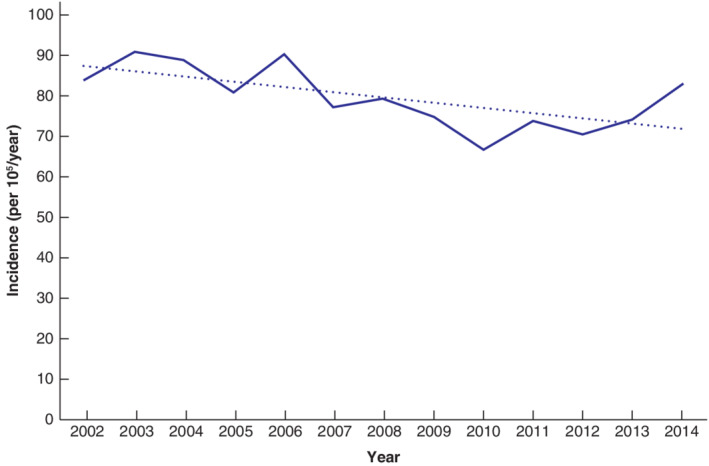
Incidence of sigmoid diverticulitis for age group 40–89 years in Iceland from 2002 to 2014 The dotted line indicates the mean of the data. The trend for the decrease in incidence of sigmoid diverticulitis was significant (*P* = 0·033, χ^2^ test for trend).

The incidence of sigmoid diverticulitis was defined as the incidence of admissions to hospital for sigmoid diverticulitis. The incidence is the number of individuals diagnosed with the disease in 1 year per 100 000 Icelanders. The denominator was calculated from the real number of individuals in each age group and time period from Statistics Iceland (https://statice.is/).

A diagnosis of sigmoid diverticulitis was given if one of the following codes appeared first in the discharge diagnosis list from a surgical ward: 562.1, K57.3, K57.5 and K57.9 (diverticular disease without perforation or abscess) or 562.1, K57.3, K57.5 and K57.9 (diverticular disease without perforation or abscess), ICD‐9 diagnosis 562.11 or ICD‐10 diagnosis K57.2, K57.4 and K57.8 (diverticular disease with perforation or abscess), or any discharge diagnosis of diverticular disease that in a later admission ended in resection of the sigmoid colon or left colon.

When the patient was discharged from a medical ward, the following codes were used to define diverticulitis: 562.11, K57.2, K57.4 and K57.8 (diverticular disease with perforation or abscess).

This categorization was tested in random populations admitted in 1995–2001, 2002–2008 and 2009–2014.

For each patient, age, sex, type of surgery (resection of the sigmoid colon or a left hemicolectomy) and the diagnostic modalities were recorded. These included: clinical diagnosis (pain and abdominal tenderness), clinical diagnosis and blood test (abdominal symptoms and raised white cell count or C‐reactive protein level), and imaging (abdominal X‐ray and CT). Sensitivity and specificity with 95 per cent confidence intervals, and Youden's index for each method were then calculated.

**Fig. 5:**
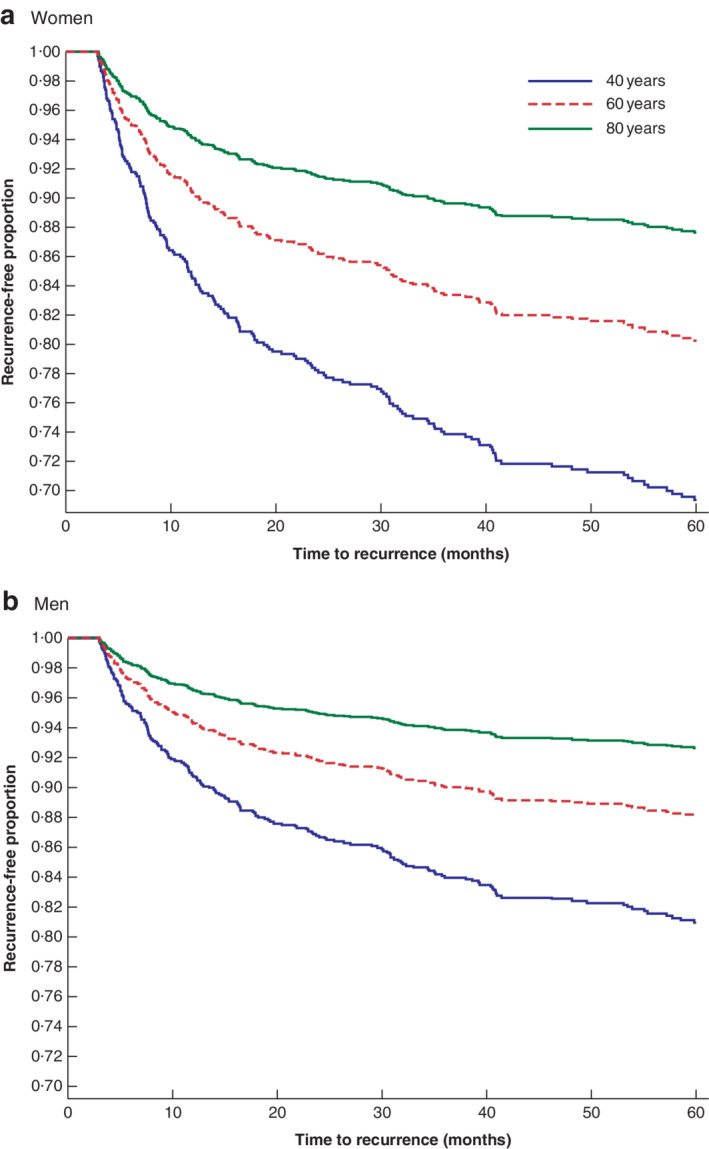
Cox proportional hazards model showing the predicted proportion of patients aged 40, 60 and 80 years with recurrence‐free first‐time diverticulitis in Iceland in 2002–2014 Mean values for patients aged 40, 60 and 80 years are shown to represent the age groups 20–49, 50–69 and 70–99 years respectively. Duration of follow‐up time was 5 years. In the Cox model, evaluation of the hazard ratio (HR) for sex was adjusted for age at first attack, and the HR for age at first attack was adjusted for sex. Age was used as a continuous variable; the HR for age was 0·97 (95 per cent c.i. 0·96 to 0·98) (*P* < 0·001). The HR for sex was 1·73 (1·28 to 2·35) (*P* < 0·001), with male sex as the reference.

**Fig. 6:**
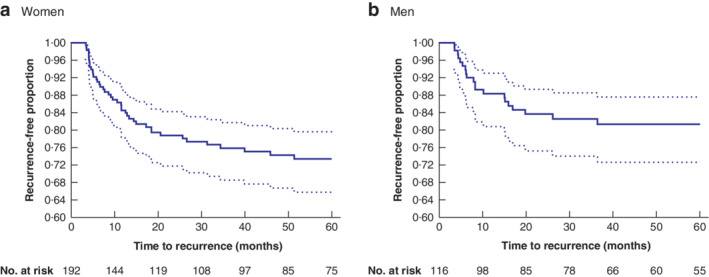
Kaplan–Meier survival estimates of recurrence‐free second attacks of diverticulitis in 2002–2014 Estimates for **a** women and **b** men with 95 per cent confidence intervals. Duration of follow‐up was 5 years.

The total number of admissions for diverticular disease and diverticulitis were registered. Patients were analysed according to sex (women *versus* men), age (at first admission) and admission year. Recurrent diverticulitis was defined as a new admission after an interval of more than 90 days from the previous episode, whereas admissions after less than 90 days were considered as the same episode.

All patients with a first admission for diverticulitis who did not have a resection (2002–2008 and 2009–2014) were considered at risk and were analysed for recurrence using survival analysis.

### Statistical analysis

Data processing was performed in Excel® 2010 and analysed using SPSS® statistics (IBM, Armonk, New York, USA). Statistical computation was performed in Stata® 13 (StataCorp, College Station, Texas, USA), setting significance at 0·05. To test for a trend over time, the χ^2^ test for trend was used. Survival analyses were conducted for the period 2002–2014 to compare recurrence for different sex and age groups. The Cox proportional hazards model was used to predict survival of patients with first‐time diverticulitis, adjusting for age at first attack and sex. The Kaplan–Meier method with 95 per cent confidence intervals was used to show disease‐free survival, censoring at date of death or date of colonic resection. The end of the study was 31 December 2014.

## Results

Between 1985 and 2014, there were 8660 hospital admissions for diverticular disease and complicated diverticulitis. Diverticulitis was diagnosed in 4746 admissions, of which 2939 were first‐time admissions in 1701 women (57·9 per cent) and 1238 men (42·1 per cent). Overall, 965 patients required a surgical resection. After the first attack, 661 patients had surgery; of the 2278 patients who were not treated by resection, 537 (23·6 per cent) had a second attack (*Fig*. *1*).

As shown in *Tables* *1*, *2*, *3*, *4* and *Figs* *2* and *3*, the incidence of admissions and resections for sigmoid diverticulitis increased from 1985 to 1995 in all age groups. The incidence was unchanged in the age groups 20–39 years (119 patients) from 2000 to 2014. However, in the 2770 patients aged above 40 years the incidence decreased after the year 1995, with a more prominent decrease in the older age groups; in particular, in patients aged 70 years or more the decrease was greater than 50 per cent. The incidence and number of sigmoid resections followed the same trend as the incidence and number of admissions for diverticulitis.

**Table 1:** Patients in 10‐year age groups with first‐time sigmoid diverticulitis admitted to hospital in Iceland in 5‐year periods from 1985 to 2014

	No. of patients with diverticulitis (*n* = 2934*)					
Age group (years)	1985–1989	1990–1994	1995–1999	2000–2004	2005–2009	2010–2014
20–29	0	3	2	8	4	10
30–39	7	13	20	29	34	34
40–49	16	47	87	118	88	85
50–59	39	74	108	137	140	139
60–69	54	116	109	96	107	148
70–79	82	116	129	124	109	77
80–89	42	68	71	64	70	55
90–99	6	8	11	11	8	11

* Five patients were excluded as aged 10–19 years.

**Table 2:** Incidence of sigmoid diverticulitis in 10‐year age groups of patients admitted to hospital in Iceland in 5‐year periods from 1985 to 2014

	Incidence of diverticulitis (per 10^5^/year)					
Age group (years)	1985–1989	1990–1994	1995–1999	2000–2004	2005–2009	2010–2014
20–29	0	0·28	0·20	0·75	0·35	0·85
30–39	0·76	1·27	1·90	2·69	3·13	3·06
40–49	2·56	6·04	9·61	11·73	7·85	7·98
50–59	7·29	14·05	17·88	18·10	15·43	13·74
60–69	12·07	23·95	22·15	19·62	18·75	20·62
70–79	29·42	37·41	36·83	31·81	25·98	18·50
80–89	32·54	48·88	46·24	36·99	34·31	22·81
90–99	28·16	32·85	44·05	38·41	24·41	27·75

**Table 3:** Number of resections for sigmoid diverticulitis in 10‐year age groups of patients admitted to hospital in Iceland in 5‐year periods from 1985 to 2014

	No. of resections for diverticulitis					
Age group (years)	1985–1989	1990–1994	1995–1999	2000–2004	2005–2009	2010–2014
10–19	0	1	0	0	0	0
20–29	0	1	0	3	0	1
30–39	3	2	4	8	5	7
40–49	3	21	27	39	32	13
50–59	11	23	46	53	60	49
60–69	21	43	50	39	42	48
70–79	29	48	48	31	39	35
80–89	7	19	11	9	15	9
90–99	2	1	1	2	3	1

**Table 4:** Incidence of resection for sigmoid diverticulitis in 10‐year age groups of patients admitted to hospital in Iceland in 5‐year periods from 1985 to 2014

	Incidence of resection for diverticulitis (per 10^5^/year)					
Age group (years)	1985–1989	1990–1994	1995–1999	2000–2004	2005–2009	2010–2014
10–19	0	0·09	0	0	0	0
20–29	0	0·09	0·00	0·28	0·00	0·09
30–39	0·33	0·20	0·38	0·77	0·46	0·63
40–49	0·48	2·70	2·98	3·85	2·89	1·22
50–59	7·29	14·05	17·88	18·10	15·43	13·74
60–69	4·78	8·96	10·16	7·97	7·36	6·69
70–79	10·28	15·35	13·71	7·89	9·47	8·41
80–89	5·42	13·66	7·27	5·20	7·25	3·73
90–99	9·39	4·11	3·67	6·98	9·15	2·52

In 1995–2014 there was a linear trend for the decrease in the incidence; *P* for trend for all ages was 0·009 and for the age group 40–89 years it was less than 0·001.

The methods used to establish the diagnosis of sigmoid diverticulitis were determined in 50 random patients in each period (1995–2001, 2002–2008, 2009–2014), as summarized in *Table* *5*. There was a radical change in the diagnostic methods used during those years. In 1995–2001, diagnosis was based on clinical diagnosis, blood tests and abdominal X‐ray in 13 patients and on CT or surgery in 18 patients, compared with CT or surgery in all patients in the other two time periods, 2002–2008 and 2009–2014. There is thus a risk of lower diagnostic accuracy in the first period, 1995–2002. The sensitivity and specificity, and Youden's index for the diagnosis of sigmoid diverticulitis was calculated for the three time periods (*Table* *6*). Sensitivity and specificity were over 90 per cent for the two latter periods. Results for the period 1995–2001 were assumed to be less reliable, and were therefore excluded for trend calculation and analysis of recurrence.

**Table 5:** Diagnostic methods used to reach the discharge diagnosis of sigmoid diverticulitis in 50 patients chosen randomly from each of the three periods

	No. of patients (*n* = 50)		
Diagnostic method	1995–2001	2002–2008	2009–2014
Surgery	9	4	11
CT	9	17	23
Abdominal X‐ray	2	0	0
Fever, WBC, CRP	5	0	0
Clinical diagnosis	6	0	0
Sigmoid diverticulitis*	31	21	34
ICD classification method†	27	18	32

* Number of patients with sigmoid diverticulitis at discharge according to information read in the patient files in 50 records chosen randomly from all admissions for diverticular disease in the three periods.

† Number of patients with sigmoid diverticulitis at discharge according to the method used in the present study, based on the ICD classification. WBC, white blood cells; CRP, C‐reactive protein.

**Table 6:** Sensitivity and specificity, and Youden's index for the method used to diagnose sigmoid diverticulitis

Period	Sensitivity (%)	Specificity (%)	Youden's index*
1995–2001	85·2 (74·9, 95·5)	100	0·85
2002–2008	94·4 (87·8, 101·1)	92·6 (84·9, 100·2)	0·87
2009–2014	93·8 (86·8, 100·8)	100	0·94

Values in parentheses are 95 per cent confidence intervals.

* Sensitivity + specificity − 1.

The incidence of first‐time diagnosis of diverticulitis (age groups 40–89 years; 1330 patients) decreased between 2002 and 2014, with a significant trend over time (*P* = 0·033) (*Fig*. *4*).

*Fig*. *5* shows the survival analysis for recurrence of sigmoid diverticulitis. The Cox model, corrected for sex and age at the first attack of diverticulitis, was used for 1426 individuals who had their first attack in 2002–2014. During a 5‐year follow‐up or to the end of the study, 197 patients had a second attack of diverticulitis. The risk of developing a second attack was greater in women than in men (*P* < 0·001). It was also greater in patients aged 20–49 years than in those aged 50–69 years, and lowest in the age groups 70–99 years (*P* < 0·001).

During 2002–2014, 63 of 308 patients with a second attack of diverticulitis (111 patients who had their first attack before 2002 and 197 who had their first attack in 2002–2014) also had a third attack during a 5‐year follow‐up or to the end of the study (*Fig*. *6*). Kaplan–Meier analysis estimated that the risk of developing a third attack was higher in women, but the number of individuals was too small for age groups to be compared.

In 2002–2014, 493 patients had resection of the sigmoid colon or a left hemicolectomy for sigmoid diverticulitis. These patients were followed up for 5 years or to the end of the study; seven men and nine women (3·2 per cent) were diagnosed with a recurrent attack of diverticulitis.

## Discussion

Between 1995 and 2014, the incidence of first‐time diverticulitis decreased in patients aged 40–89 years in Iceland. The incidence of colonic resection for diverticulitis also decreased, strengthening the validity of the present results. The decrease was higher in the older age groups; for patients aged 70 years and above with a first attack of sigmoid diverticulitis, the reduction in incidence was greater than 50 per cent. However, the number of individuals diagnosed with diverticulitis remained high because of the increasing age of the population. Young age and female sex were associated with a higher recurrence rate of diverticulitis.

Another study^10^, which used a comparable definition of diverticulitis, reported an increased incidence of diverticulitis between 1988 and 2012. After the introduction of CT to investigate acute pain in the abdomen in the 1990s, more patients have been diagnosed with diverticulitis that earlier would probably have been categorized as non‐specific abdominal pain. This may explain the increase in the number of patients discharged with a diagnosis of diverticulitis, but not the decrease in the incidence of diverticulitis after 1995. Another explanation is a general improvement in the health status of the older Icelandic population. The incidence of diverticulitis among those aged 70 years or more decreased by more than 50 per cent from 1995 to 2014.

The increased incidence of diverticulitis from 1985 to 1995 and the subsequent sharp decline appears to indicate an epidemic of the condition. One possible explanation is a change in lifestyle habits, including cigarette smoking, which affected 60 per cent of the adult population in Iceland in the 1960s, but dropped to 11·5 per cent in 2015^15^, in accordance with a previous study^7^ showing that smoking causes an increased incidence of complicated diverticular disease of the sigmoid colon.

In the present study, the rate of recurrent diverticulitis was higher in patients aged 20–49 years than in those aged 50–69 years, and lowest in patients aged 70–99 years. It was also higher in women than in men. These findings are supported by the results of the study from Olmsted County in Minnesota, 1980–2007^6^, in which a higher risk of recurrent diverticular disease was observed in patients who had their first attack early in life than in those with a first attack later in life, and in women compared with men.

There are no reports from the same time interval on recurrence of diverticulitis after resection. However, in an earlier report^16^ the recurrence rate was 7–10 per cent after sigmoid resection for diverticulitis. The lower recurrence rate reported here can be attributed to two factors: the length of follow‐up limited to 5 years, and the definition of recurrence as that occurring within 90 days of surgery.

The present results show that, over the years, the incidence of diverticulitis was unchanged in the young but reduced in the older population, and the risk of recurrence in younger individuals was higher than that in the elderly, suggesting a different aetiology for these groups. These differences could have an underlying genetic influence, but more investigation is needed. The sex difference in the recurrence of diverticulitis supports the previously proposed hypothesis^17^ that the aetiology of diverticulitis is different in men and women. These differences in incidence for different age groups and sexes could relate to the different lifestyles of young *versus* older people, and of men *versus* women.

## Disclosure

The authors declare no conflict of interest.
